# Effect of Cellulose Nanofibers’ Structure and Incorporation Route in Waterborne Polyurethane–Urea Based Nanocomposite Inks

**DOI:** 10.3390/polym14214516

**Published:** 2022-10-25

**Authors:** Izaskun Larraza, Julen Vadillo, Tamara Calvo-Correas, Alvaro Tejado, Loli Martin, Aitor Arbelaiz, Arantxa Eceiza

**Affiliations:** 1 Materials + Technologies’ Research Group (GMT), Department of Chemical and Environmental Engineering, Faculty of Engineering of Gipuzkoa, University of the Basque Country, Pza Europa 1, 20018 Donostia-San Sebastian, Spain; 2 IPREM-Equipe de Physique et Chimie des Polymères, UMR CNRS 5254, Université de Pau et des Pays de l’Adour, Hélioparc 2, Av. Pdt Angot, CEDEX 09, 64053 Pau, France; 3 CIDETEC, Basque Research and Technology Alliance (BRTA), Po. Miramon 196, 20014 Donostia-San Sebastian, Spain; 4 Department of Environmental Engineering, Faculty of Engineering of Vitoria-Gasteiz, University of the Basque Country, Nieves Cano Street, 12, 01006 Vitoria-Gasteiz, Spain; 5 TECNALIA, Basque Research and Technology Alliance (BRTA), Area Anardi 5, 20730 Azpeitia, Spain; 6 Macrobehavior-Mesostructure-Nanotechnology, General Research Service (SGIker), Polytechnic School, University of the Basque Country, Plaza Europa 1, 20018 Donostia-San Sebastián, Spain

**Keywords:** waterborne polyurethane–urea, 3D printing, cold extrusion, cellulose nanofibers, bioinks

## Abstract

In order to continue the development of inks valid for cold extrusion 3D printing, waterborne, polyurethane–urea (WBPUU) based inks with cellulose nanofibers (CNF), as a rheological modulator, were prepared by two incorporation methods, *ex situ* and *in situ*, in which the CNF were added after and during the synthesis process, respectively. Moreover, in order to improve the affinity of the reinforcement with the matrix, modified CNF was also employed. In the *ex situ* preparation, interactions between CNFs and water prevail over interactions between CNFs and WBPUU nanoparticles, resulting in strong gel-like structures. On the other hand, *in situ* addition allows the proximity of WBPUU particles and CNF, favoring interactions between both components and allowing the formation of chemical bonds. The fewer amount of CNF/water interactions present in the *in situ* formulations translates into weaker gel-like structures, with poorer rheological behavior for inks for 3D printing. Stronger gel-like behavior translated into 3D-printed parts with higher precision. However, the direct interactions present between the cellulose and the polyurethane–urea molecules in the *in situ* preparations, and more so in materials reinforced with carboxylated CNF, result in stronger mechanical properties of the final 3D parts.

## 1. Introduction

Three-dimensional printing additive manufacturing allows for the obtaining of complex and personalized 3D objects while reducing the waste produced during the processing of materials [1,2]. In recent years, it has gained great popularity and, thus, the search for new materials valid for this processing method is consistently growing [3]. Among 3D printing techniques, direct ink writing (DIW) and specifically cold extrusion offer further advantages, such as the possibility of obtaining ready-to-use parts without the need for high temperatures or further processing [4,5], which is of special interest for the biomedical field, where the possibility of working at low processing temperatures and the lack of additional components or steps for shape stabilization could be extremely beneficial. However, inks for cold extrusion 3D printing must meet very specific rheological properties in order to allow good printability and show good shape fidelity [6]. For this, inks must show shear-thinning behavior and defined but not too high yield points in order to allow extrusion but retain the given shape.

Polyurethane–ureas are polymers formed from the reaction of an isocyanate group with a hydroxyl group, resulting in a urethane group and the formation of an urea by reaction of isocyanate and amine groups. They are block-copolymers usually formed by two incompatible segments, the soft segment, formed by a high molecular weight polyol, and the hard segment, formed by an isocyanate and chain extender, low molecular weight alcohol or amine [7]. Polyurethane–ureas are polymers whose properties can be easily customized, making their field of application extremely wide [8]. Moreover, due to growing environmental awareness, there is a surging need to substitute conventional materials with greener options. In this regard, waterborne polyurethane–ureas (WBPUU) avoid the use of volatile organic compounds in their synthesis, and by substituting conventional precursors with reactants obtained from natural sources, environmentally friendly polymers can be obtained [9], agreeing with the climate of concern towards the environmental state of the planet. The easy customizability of these polymers, together with the easy personalization of produced parts offered by 3D printing, make them a great pairing in order to expand their use in many diverse applications, such as tissue engineering or cartilage regeneration [10].

However, waterborne polyurethane–urea dispersions often show extremely low viscosity, and overall, poor rheological behavior for DIW. In order to adapt WBPUU for DIW, first, their rheological properties must be modulated. The rheological behavior of the inks can be modified by different methods, one of the most common ones being the addition of nano-entities, such as nano-cellulose.

Cellulose is the most abundant renewable biopolymer on Earth, is found in the cell wall of plants and is the primary component of lignocellulosic biomass [11,12]. It is a linear homopolymer composed of D-glucose units bonded together by β-1,4-glycosidic bonds. Cellulose nano-entities were extensively studied [13,14,15] and, among many other uses, have shown good potential as rheological modulators for DIW. Vadillo et al. [16,17] studied the effect of the content and addition method of cellulose nanocrystals (CNC) to a PCL–PEG-based WBPUU matrix and observed that CNC were able to modulate rheological behavior improving the printability of the inks. Markstedt et al. [18] added cellulose nanofibers (CNF) to an alginate matrix to obtain 3D-printed cartilage tissue structures with good shape fidelity. Olmos et al. [19] observed that the addition of CNF to a sodium alginate matrix not only modulated the inks’ rheology, resulting in parts with better shape fidelity but also allowed for the controlled release of drugs.

Moreover, in previous studies, the effect of the modification of cellulose nanofibers by the introduction of carboxylic groups was studied. Modified CNF when added to a hydrophobic matrix can help prevent the formation of agglomerations of nano-cellulose particles in the polymeric matrix [15,20,21]. Carboxylated CNF have shown better affinity with a waterborne polyurethane–urea matrix, resulting in more strongly reinforced composites [22].

In this work, the possibility to prepare waterborne polyurethane–urea-based inks valid for DIW 3D printing was studied. In order to modulate ink properties for a good printing process and for improving the final properties of the 3D-printed parts, cellulose nanofibers were added to the inks. Moreover, the effect of the modification of cellulose on the inks was also studied by the use of carboxylated nanofibers. The effect of the type of reinforcement, content and incorporation route on the properties of the materials was studied regarding the rheology of the inks and properties of the final parts, and the possibility of using these materials in DIW was assessed.

## 2. Materials and Methods

The difunctional polyol obtained from renewable resources used in the polyurethane–urea synthesis (Priplast 3192^®^, M_w_ = 2000 g mol^−1^) was purchased from Croda. Isophorone diisocyante (IPDI, DESMODOUR I) was kindly supplied from Covestro and ethylene diamine (EDA) was provided by Fluka. 2,2-Bis(hydroxymethyl)propionic acid (DMPA, Aldrich) and triethylamine (TEA, Fluka) were used as internal emulsifiers and neutralizing agents, respectively. Dibutyltin dilaurate (Aldrich) was used as catalyst.

For cellulose nanofiber preparation, standard bleached hardwood kraft pulp (bHKP), supplied by local paper mill, was used. Sodium metaperiodate (NaIO_4_), sodium chloride (NaCl), hydrogen peroxide (H_2_O_2_), sodium hydroxide (NaOH) and sodium chlorite (NaClO_2_) employed during the carboxylation process were purchased from Scharlab. Moreover, a commercial cellulose, CNFr, was employed as reference and for inks’ CNF content optimization. For this freeze-dried cellulose nanofibers provided by the University of Maine (Lot. 9004-34-6) were used.

### 2.1. Synthesis Process and Obtaining of CNF

The synthesis of the polyurethane–urea and the production and carboxylation of the cellulose nanofibers were carried out following the procedures optimized and explained in previous works [22,23].

Briefly, a waterborne polyurethane–urea, with a molar ratio of polyol/DMPA/IPDI/EDA of 1/1.1/3.5/0.6, was synthesized using a two-step polymerization process. In the first step, the prepolymer, formed by the polyol, the diisocyanate and the catalyst, was synthesized and was left to react for 5 h at 100 °C under constant mechanical stirring. The reaction was then cooled to 50 °C and the DMPA neutralized with TEA was added and maintained under agitation for 1 h. In the second step of the synthesis, the phase inversion was carried out by dropwise addition of deionized water under vigorous stirring at room temperature. Finally, the chain extension was carried out at 35 °C for 2 h. An aqueous dispersion of WBPUU with a solid content of 33 wt% was obtained.

CNF were obtained from bHKP sheets, which were cut into pieces and swelled on water for 24 h. The mixture was then dispersed by mechanical agitation until it became homogeneous. The prepared suspension was passed through a Masuko Supermass Colloider (MKZA10-15J) until no microstructures were present and cellulose nanofibers were obtained, for which 10 passes were necessary. The isolated nanofibers were denominated as CNF0.

For the carboxylation of the CNF, a sequential periodate–chlorite oxidation treatment was carried out parting from the prepared suspension [22,24]. In the first step of the treatment, the suspension was mixed with NaIO_4_ and NaCl, and was left to react under total darkness for 2 h. In the second step, NaClO_2_, NaCl and H_2_O_2_ were added, and the mixture was left to react for 2 h. The pH was maintained between 4.2 and 4.5, using NaOH. Afterward, the reaction mixture was filtered and the obtained fibers washed repeatedly. Lastly, nanofibers were obtained by disintegration using a Masuko Supermass Colloider, after 8 passes. The obtained carboxylated cellulose nanofibers were denominated CNF1.

### 2.2. Ink Preparation

For the preparation of the WBPUU/CNF inks, three different cellulose nanofiber systems were employed, untreated cellulose nanofibers (CNF0), carboxylated cellulose nanofibers (CNF1) and commercial cellulose nanofibers (CNFr). Inks were prepared by two incorporation methods, *ex situ* and *in situ*. *Ex situ* addition was carried out by addition of dried CNF to the already synthesized WBPUU dispersion and homogeneity was achieved by vigorous mechanical stirring, using an ultraturrax homogenizer (Polytron PT 2500E, KINEMATICA). Stirring was carried out in an iced bath, in order to avoid high temperatures and degradation of the systems. For *in situ* preparation, reinforcements were added dispersed in water during the phase inversion step of the synthesis of the polyurethane–urea.

For *ex situ* preparations, inks with 2 and 3 wt.% of CNF, regarding the total weight of the inks, were prepared with CNF0 (2CNF0_EX_ and 3CNF0_EX_) and CNF1 (2CNF1_EX_ and 3CNF1_EX_). For *in situ* preparations, first, CNF content was optimized using CNFr, inks with 2 and 3 wt.% of CNFr were named 2CNFr_IN_ and 3CNFr_IN_, respectively. After quantity optimization, inks with 3 wt.% of CNF0 and CNF1 were prepared *in situ* and named 3CNF0_IN_ and 3CNF1_IN_, respectively. Designation and quantities used in the preparation of WBPUU/CNF nanocomposite inks are shown in Table 1.

### 2.3. Three-Dimensional Printing

The prepared inks were used in DIW 3D printing. For this, an adapted Tumaker Voladora printer was used. Printing process was carried out at room temperature at a printing speed of 6 mm·s^−1^, using a needle with a 0.8 mm internal diameter. The material was printed on a Teflon surface and obtained printed parts were right away frozen in order to freeze-dry them for the preparation of scaffolds.

**Figure 1:**
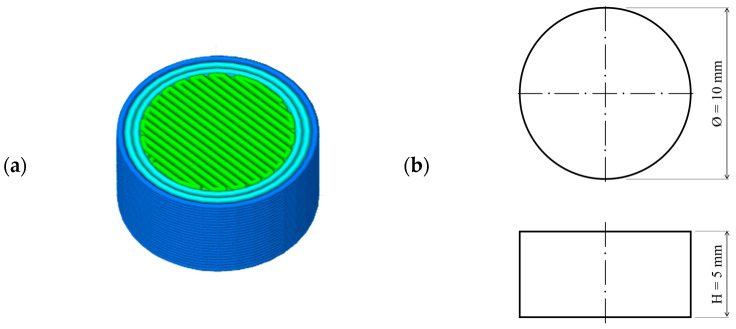
(**a**) Three-dimensional model of imported cylinder design and (**b**) dimensions.

For printing design, a cylinder was chosen, with a diameter of 10 mm and a height of 5 mm (Figure 1). Printed parts were named “3D-X”, where “X” is the name of their corresponding inks.

### 2.4. Characterization

#### Rheological Properties of the Prepared Inks

Rheological characterization of WBPUU dispersion and inks was performed using a Haake Viscotester iQ (Thermo Scientific). Tests were performed at 25 °C and employed geometry was chosen depending on the tested material. For low-viscosity materials, a coaxial cylinder geometry was used (CC25 DIN/Ti adapter), with a piston radius of 12.54 mm and a ring gap of 1.06 mm. For inks with higher viscosities, a plate–plate geometry was used (P35/Al adapter), where plates with a diameter of 35 mm were employed and a working gap of 1 mm was set.

For flow tests, shear rate sweeps from 0.2 to 1000 s^−1^ were performed. For yield point determination tests, dynamic oscillatory tests were performed in a shear stress range of 10 up to 10,000 Pa, depending on the tested sample. Last, structure recovery tests were performed in a three-stage experiment, in order to simulate the direct ink writing process and the subsequent modulus/rigidity recovery. In this test, viscosity values were measured at a shear rate of 0.2 s^−1^ during 100 s, followed by a shear rate of 100 s^−1^ for 100 s in the second step and ending with the same initial condition for the third step.

Flow index, n, of each ink was calculated from Power Law (Equation (1)). Yield point was determined as the point of deviation of G’ from linearity, as proposed by Cyriac et al. [25].

(1) $$\eta (\dot{\gamma })=K{\dot{\gamma }}^{n-1}$$

where η is the viscosity (Pa·s); $\dot{\gamma }$ is the shear rate (s^−1^); K is the consistency index (Pa·s^n^); and n is the flow index (dimensionless).

Flow point, on the other hand, was measured as the crossover point for G’ and G’’. For the determination of the structural recovery capacity of the inks, a ratio of the ink’s viscosity after the high shear rate process and before it was calculated, as expressed in Equation (2).

(2) $$\mathrm{Structural}\;\mathrm{Recovery}=\frac{\eta_{\mathrm{AS}}}{\eta_{\mathrm{BS}}}\cdot 100$$

where η_BS_ is the viscosity of the material before the high shear rate process and η_AS_ is the viscosity after the high shear rate process; in both cases, the viscosity was measured after 80 s from the beginning of that part of the test.

### 2.5. Characterization of 3D-Printed Composites

#### 2.5.1. Scanning Electron Microscopy

The morphology of the 3D-printed and freeze-dried scaffolds was observed by Scanning Electron Microscopy (SEM), using Field Emission Gun Scanning Electron Microscopy (FEG-SEM) Hitachi S-4800N, at a voltage of 5 kV.

Prior to the test, and in order to analyze the cross-section of prepared scaffolds, the samples were cryofractured in liquid nitrogen and sputter coated with a thin layer of gold (∼10 nm) in an Emitech K550X ion sputter.

#### 2.5.2. Differential Scanning Calorimetry

The thermal properties of the freeze-dried polyurethane–urea and 3D-printed samples were determined by Differential Scanning Calorimetry (DSC) using a Mettler Toledo DSC 3+ equipment provided with a robotic arm and an electric intracooler as refrigerator unit. Samples (5–10 mg) were encapsulated in aluminum pans and heated from −65 to 200 °C at a scanning rate of 10 °C min^−1^ in nitrogen atmosphere. From the heating thermograms, order–disorder transition temperature, related to the short-range ordering of the hard segment of polyurethane–urea (T_HS_), and enthalpy (ΔH_HS_), as the maximum of the peak and the area below the peak, respectively, as well as glass transition temperature (T_g_), as the inflection point of the heat capacity change, were determined.

#### 2.5.3. Compression Tests

The mechanical properties of the 3D-printed samples were studied by compression tests. Tests were carried out at room temperature using an Instron 5967 universal testing machine provided with a 500 N load cell. Compression force was applied in the normal direction from the layer-by-layer printing. Cylindrical samples (Ø = 10 mm and h = 5 mm) were compressed to a fixed deformation of 60% at a crosshead speed of 10 mm·min^−1^.

The compression modulus was calculated as the slope of the stress–strain curve at low deformations, the stress was measured at 60% of strain and densification strain was determined at the intersection point between the stress plateau and a line extrapolated from the densification line. Moreover, specific Young modulus values were measured as the ratio between each sample’s Young modulus and its density. The density of the 3D-printed and freeze-dried samples was calculated as the ratio between their measured weight and volume. Compression values were averaged for five specimens.

## 3. Results and Discussion

### 3.1. Rheological Characterization of the Inks

The rheological behavior of the prepared inks was characterized for a preliminary study of the printability and shape fidelity that they will show when used in direct ink writing 3D printing.

**Figure 2:**
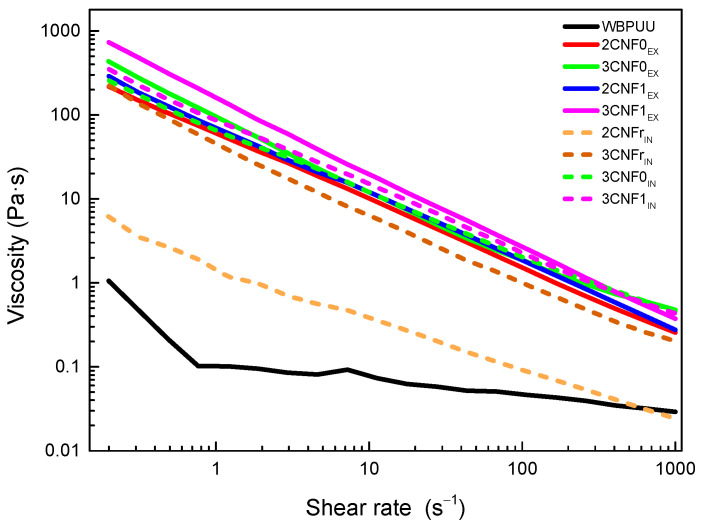
Viscosity flow curves of neat WBPUU and WBPUU/CNF inks.

Flow tests were carried out in order to study the effect of the shear rate on the viscosity of the prepared nanocomposite inks. The obtained flow curves are shown in Figure 2 for WBPUU and CNF-containing inks. Moreover, viscosity values at a shear rate of 0.2 and 100 s^−1^, as well as at the shear rate on the wall of the nozzle (γ_nz_), calculated with Equation (3), and flow index, n, are summarized in Table 2.

When studying the rheological behavior of a shear-thinning ink for 3D printing, it is worth noting that its non-Newtonian behavior will cause the flow on a capillary to deviate from a parabolic velocity profile [26]. Thus, for the correct calculation of the shear stress that the ink will be subjected to, the equation proposed by Li et al. [27] (Equation (3)), containing the necessary adjustments, can be used.

(3) $${\dot{\gamma }}_{n}^{n}={\left[\frac{V\cdot R^{2}}{\left(\frac{n}{3n+1}\right)\left(R^{\frac{3n+1}{n}}\right)}\right]}^{n}\cdot r$$

where ${\dot{\gamma }}_{n}$ is the shear rate the ink is submitted to at the nozzle; n is the flow index calculated from Power Law; V is the printing speed; r is the distance located between the center of the nozzle and its radius; and R is the radius of the nozzle.

As can be observed from flow curves, all systems showed shear-thinning behavior, which is ideal for DIW [28,29], with all inks presenting n values corresponding to pseudo-plastic fluids (n < 1). However, differences in the viscosities of the systems depending on the type of reinforcement, content and addition route were evident.

Regardless of the incorporation route, the addition of CNF to the polyurethane dispersion resulted in a very intense increase in the viscosity of the systems, in agreement with the reported behavior after the addition of cellulose [16,30,31,32,33]. The obtained results indicated that interactions between water molecules and nano-cellulose were created. Moreover, the increase in viscosity was directly related to the CNF content (Table 2), once again agreeing with the literature reports [34,35,36,37]. The higher viscosities shown by nanocomposite inks containing 3 wt.% of CNF may result in 3D-printed parts with better shape fidelity, thanks to their lower tendency to flow at low shear rates, i.e., at rest.

Regarding the type of cellulose used, the results show that carboxylated cellulose nanofibers resulted in inks with higher viscosity. The higher viscosities shown by CNF1-containing inks may be due to the carboxylic groups facilitating the formation of interactions between water molecules and nano-cellulose, as also observed in the literature [33,34,38]. On the other hand, the used reference cellulose system, due to its complete lack of carboxylic groups, resulted in inks with lower viscosities.

**Figure 3:**
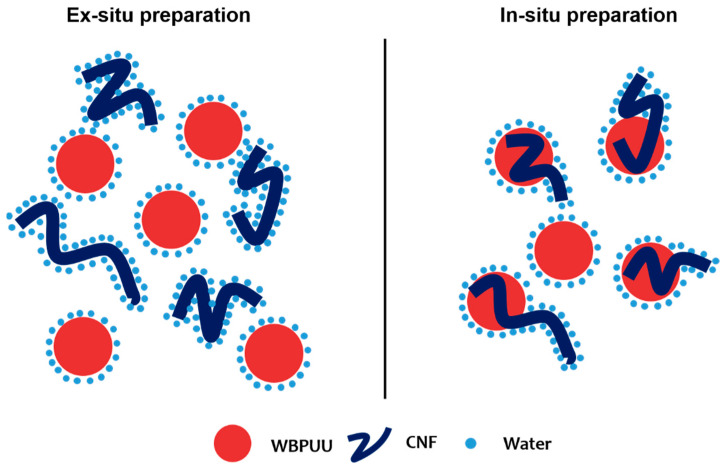
Schematic representation of interactions taking place through different preparation methods.

Regarding the incorporation method, it was observed that *in situ* addition resulted in inks with lower viscosity values than their *ex situ*-prepared homologs. In *ex situ* addition, the added nanostructures were not able to easily approach the already synthesized and stable particles of WBPUU due to repulsive forces between the matrix and the reinforcement [39], and CNF were more likely to interact with water instead, which allowed for a better gel formation. With *in situ* addition, on the other hand, the addition of the nano-reinforcements during the particle formation step and the strong agitation allowed the proximity of the components and may have resulted in nanostructures partially placed inside the WBPUU particles, as seen for cellulose nanocrystals [40], which favored matrix/reinforcements interactions and hindered interactions with water. A schematic representation of this can be observed in Figure 3. Moreover, despite the phase inversion and chain extension steps of the synthesis being carried out at low temperatures in order to favor NCO and NH_2_ reaction [41], it is possible that with *in situ* addition, some OH groups of the nano-entities may have reacted with NCO and formed chemical bonds between nano-cellulose and polyurethane–urea molecules.

Overall, it was observed that cellulose nanofibers are good viscosity modulators. The lower viscosity of *in situ* inks at the printing shear rate will allow for a better flow and will facilitate the extrusion process. However, the higher viscosity values at a rest-like state shown by the *ex situ*-prepared inks will enhance the shape fidelity of the printed structures.

**Figure 4:**
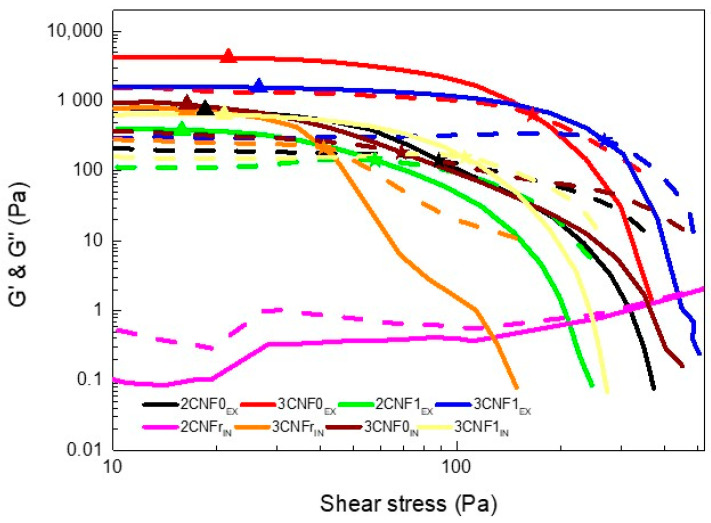
Storage (solid line) and loss (dotted line) moduli as a function of shear stress and (▲) yield and (★) flow points of WBPUU/CNF inks.

Spectro-mechanical analyses were performed in order to determine the yield and flow points of the inks and to study the dependence of their storage and loss moduli on the applied shear stress. The storage and loss moduli vs. shear stress curves are shown in Figure 4. Yield point and flow point values are summarized in Table 3.

As can be observed, the 2CNFr_IN_ ink did not show a gel-like behavior, with G’’ values being higher than G’ values throughout the stress sweep, for this system, the amount of cellulose added was not enough to form a gel structure. For this reason, this ink was discarded for use in 3D printing since it did not adjust to the rheological requirements. All other inks showed G’ > G’’ values at low shear stress, proving a gel-like behavior; however, this changed when the applied shear stress increased.

Overall, the inks showed relatively low yield points. The *ex situ*-prepared composites containing 3 wt.% of cellulose showed slightly higher yield points, suggesting the higher amount of cellulose in the system resulted in a resistance to flow, agreeing with the literature results [16,30,31]. Regarding the flow point, for the *ex situ*-prepared inks, it can be observed that it was directly related to the content and type of cellulose used, being higher for inks with higher contents of CNF, as seen in previous work with cellulose nanocrystals [16], as well as for systems with carboxylated CNF at the highest content. The higher amount of cellulose and interactions in these systems were able to maintain a more stable network at higher shear stress. For the *in situ*-prepared inks, similar yield points were observed regardless of the type of CNF used. However, when analyzing the flow point, it can be observed that for CNFs with higher carboxylation degrees, higher flow point values were measured, once again suggesting the formation of more interactions when carboxylated cellulose is used.

Regarding the effect of the reinforcement incorporation route, it can be observed that less-gelled structures were formed when components were added using the *in situ* method. The *ex situ* method favoring the additive’s interactions with water resulted in stronger gel structures, which is in agreement with the viscosity test results.

The defined yield point shown by all gelled systems (all inks except 2CNFr_IN_) may allow for a good shape fidelity of the inks when used in 3D printing [16,42]. Moreover, the reinforcement effect supplied by the addition of CNF and the corresponding increase in the storage modulus will benefit shape fidelity due to a higher capacity to maintain the given shape without collapsing with the weight of the layers deposited on top.

**Figure 5:**
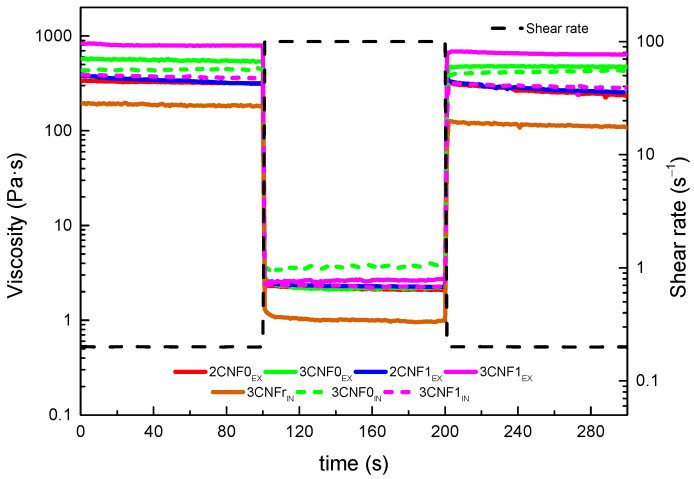
Structure recovery tests of WBPUU/CNF inks.

The structural integrity, thixotropic behavior and potential shape fidelity of the inks were studied by recovery tests, in which the capacity of a material to recover its initial viscosity after a high shear rate state was analyzed. Ideal inks for DIW should show low viscosity when subjected to a shear force but will quickly recover high viscosity when this force is removed [27]. The obtained recovery curves are shown in Figure 5. The recovery capacity of each system was calculated using Equation (2), considering their viscosity after 80 s of the low shear rate, simulating a state of rest. The calculated recovery values for all inks are shown in Table 3.

Overall, it can be observed in Figure 5 that when a shear rate of 100 s^−1^ was applied, the viscosity of all inks drastically dropped to low values, which would allow for a good flow during the printing process. Moreover, it can be seen that when this shear force was removed, the viscosity of the systems immediately went back to higher values.

For composite inks, it can be observed that the *ex situ* preparation method resulted in higher recovery capacity than the *in situ* preparations. The interactions present in the *ex situ* preparations and damage during the high shear rate step could recover more easily and faster than interactions present with the *in situ* preparations. The lower recovery capacity observed for these systems may later result in poorer shape fidelity of the printed parts due to a higher likelihood to flow suggested by the lower viscosity they showed after being under high shear rates. For the *ex situ* composites, a good recovery capacity was observed for all systems, with values around 80%, which suggested a good shape fidelity after the printing process [43].

Overall, it can be concluded that the addition of the new components to the WBPUU dispersion successfully resulted in the formation of gel structures, with significantly increased viscosity. The obtained results point to a promising extrusion process by the shear-thinning behavior of the systems and their ability to flow under applied pressure, and to a potentially good shape fidelity by their defined yield points and their structural recovery capacity.

### 3.2. Characterization of the 3D-Printed Parts

**Figure 6:**
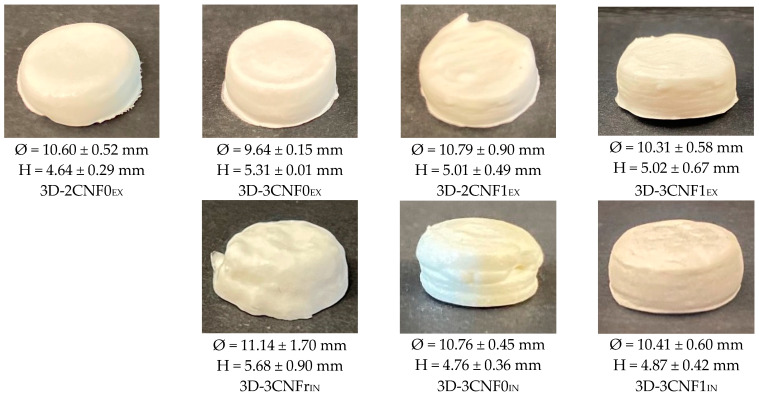
Photographs of 3D-printed cylinders from WBPUU/CNF inks.

Inks showing gel-like behavior were used in a DIW printer to produce 3D-printed objects, whereas WBPUU dispersion and 2CNFr_IN_ were discarded since they did not meet the rheological requirement for DIW. The obtained 3D-printed parts are shown in Figure 6.

As can be observed, the differences in rheology translated in different printing capacities and precision, and the content, type and incorporation route of the reinforcement played a part in the printability of the materials, with systems with stronger gel-like behavior showing better results, as previously reported [6,44]. Overall, all systems showed a good extrusion process thanks to their shear-thinning behavior. However, the *ex situ*-prepared inks sometimes caused nozzle obstruction, which was attributed to the presence of agglomerations. For the *in situ* preparations, the addition of CNF under strong agitation during the dispersion formation phase allowed for higher homogeneity.

It can be observed that the content of cellulose affected the shape fidelity shown by the inks, with inks containing a higher amount of cellulose showing better shape fidelity, agreeing with the literature reports [16,45]. The ink 2CNF0_EX_, due to its high tendency to flow and lower storage modulus, did not allow the material to retain its printed shape. On the other hand, 3CNF1_EX_ showed the best shape fidelity and was able to support layers upon layers without flowing or collapsing due to the good rheological behavior it presented. For the *in situ*-prepared CNF-containing inks, it was observed that their weaker gel-like behavior and higher tendency to flow resulted in a slightly poorer shape fidelity when compared with the *ex situ* inks. The material with the lowest viscosity and lower yield point, 3CNFr_IN_, showed the worst shape fidelity, whereas 3CNF1_IN_, with the highest viscosity and yield point values among the *in situ* preparations showed the best shape fidelity.

**Figure 7:**
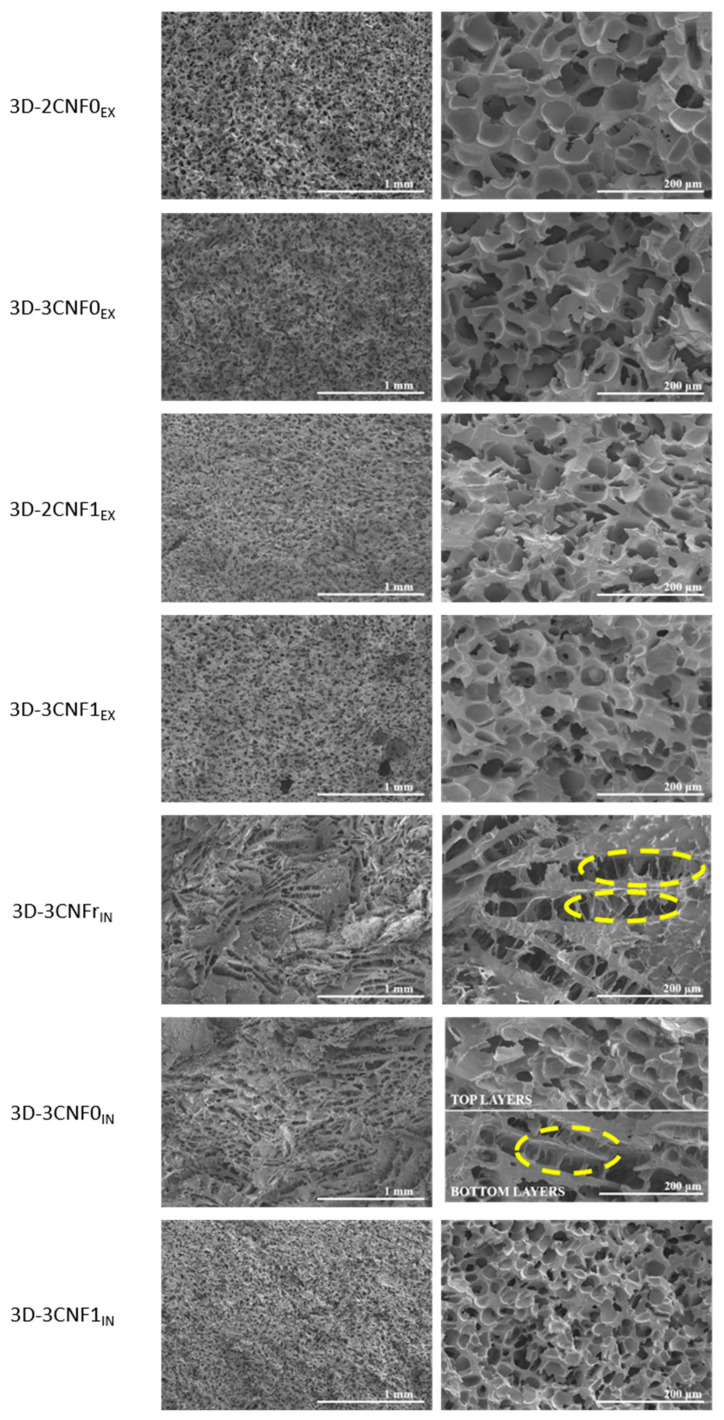
SEM images of 3D-printed parts from CNF-containing inks. (**left**) ×50 and (**right**) ×250 magnification. (Yellow circles show collapsed cell walls).

The morphology of the printed parts was studied by SEM micrographs at different magnifications of a cryofractured cross-section (Figure 7). It can be observed that the morphology of the 3D-printed parts was strongly influenced by both the composition and preparation method of the inks. Pore diameters were measured using ImageJ software and averaged out of 50 measurements.

The 3D-printed parts showed clear morphological differences depending on the ink preparation method and directly related to the rheological behavior of the inks. Regarding the *ex situ* preparations, all systems showed a similar morphology, composed of spherical pores and with high homogeneity and with evenly distributed pores. However, some differences in pore size could be observed, depending on the composition of the ink. Pore diameters of 54.7 ± 10.7, 54.1 ± 9.1, 45.0 ± 9.8 and 47.9 ± 6.1 µm were measured for 3D-2CNF0_EX_, 3D-3CNF0_EX_, 3D-2CNF1_EX_ and 3D-3CNF1_EX_, respectively. The results suggested that the pore size was not influenced by the nano-cellulose content of the ink but seemed to be influenced by the surface modification of the fibers. The printed parts containing carboxylated nano-cellulose, CNF1, showed slightly lower pore size than their CNF0 counterparts. The higher capacity of carboxylated CNF to form interactions with WBPUU and water seemed to result in smaller pores. On the one hand, the interaction between CNF and PUU as seen for chemical crosslinking can reduce pore size [46], on the other hand, interactions between CNF and water result in a smaller amount of free water in the systems, for which the elimination process was reported to result in more porous structures [47]. Moreover, the high homogeneity and spherical pore morphology shown by these systems were a result of the rheological properties shown by their corresponding inks being able to support the weight of the upper layers [16].

For 3D-printed parts obtained from the *in situ*-prepared inks, however, a different morphology was observed. In these systems, more heterogeneous structures were found. A pore-dominated structure was still present but these pores no longer showed a spherical shape, instead elongated pores were observed. The weaker gel behavior shown by the *in situ*-prepared inks was not able to support the weight of the layers on top [16,48,49]. As a result, collapsed cell walls can be observed when analyzing the morphology (circled in yellow in Figure 7), a common problem in the fabrication of scaffolds [50]. This effect was directly related to the viscosity, yield point and storage modulus shown by their corresponding inks, with 3CNFr_IN_ showing the lowest values and 3D-3CNFr_IN_ showing the most collapsed structures. The ink 3D-3CNFr_IN_ showed elongated pores with a height of 54.5 ± 15.6 µm and a length of 322.1 ± 38.2 µm. On the other hand, the higher values of viscosity, yield point and storage modulus shown by 3CNF1_IN_ were reflected in a more homogeneous structure and the recovery of spherical pores. For 3D-3CNF1_IN_ spherical pores with an average diameter of 36.3 ± 11.1 µm were observed. However, for 3D-3CNF0_IN_ both types of pores can be observed, being the spherical pores (Ø = 46.8 ± 15.0) the predominant shape of the top layers and the elongated pores (H = 43.5 ± 9.9 and L = 247.8 ± 52.2) the most present on the bottom layers. Though, 3CNF0_IN_ did not present sound enough rheological properties to support upper layers, it also did not flow as easily as 3CNFr_IN_. Therefore, for this system, crushed structures were obtained in the bottom zone of the structure since it was not capable of supporting the weight of the upper layers, but the top layer, without that added weight, was able to maintain a steady structure.

Changes in the thermal transitions of the 3D-printed parts were studied by DSC. The measured T_gSS_, T_HS_ and ΔH_HS_ values are summarized in Table 4. No significant changes can be observed regarding the glass transition temperature of the soft segment, with all systems showing a T_g_ value of around −50 °C. However, some changes are observed in the DSC curves when studying the hard segment short-range ordering transition of the materials.

It can be observed that increasing the nano-cellulose content and the use of carboxylated nanofibers increased the transition temperature and enthalpy values of the material. This fact suggests the formation of interactions between the urethane and urea groups of the PUU hard segment and the CNF and, therefore, a more difficult breaking process of the short-range ordered structures, which is in agreement with other studies based on cellulose-reinforced polyurethanes [51,52]. For 3D parts from *in situ* preparations, an even higher increase in the enthalpy is observed, attributed to the favored polymer/reinforcements interactions by this incorporation method, as well as to the formation of chemical bonds. It can be observed that composites reinforced with the non-commercial, CNF0 and CNF1, showed higher values than those containing the commercial nano-cellulose. The carboxylation of CNF1 resulted in a better affinity with the polyurethane and, in consequence, in a higher amount of fiber/polymer interactions than in systems with commercial nano-cellulose.

**Figure 8:**
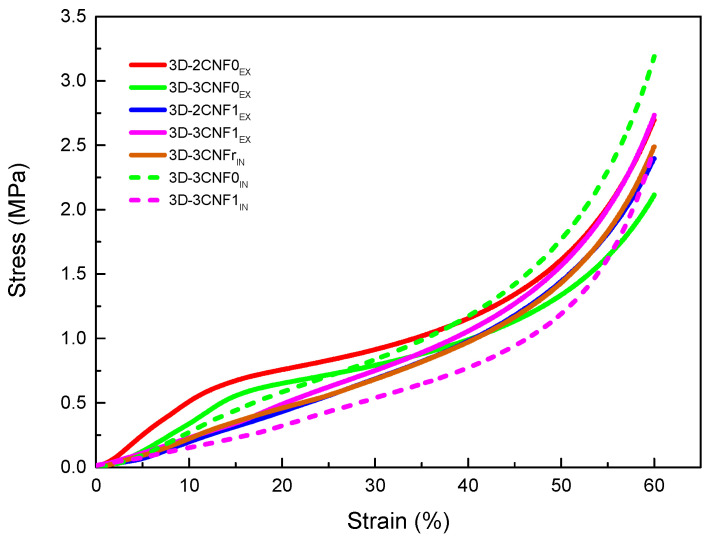
Stress/Strain curves from compression tests for WBPU/CNF 3D-printed parts.

In order to study the mechanical behavior of the printed parts, compression tests were carried out. The obtained results are summarized in Table 5, where density, specific Young modulus, stress at 60% strain and densification strain values are shown. The stress/strain curves obtained for the CNF-containing 3D-printed parts are shown in Figure 8.

All printed parts showed a typical three-step compression behavior, divided into the elastic, the plastic and the densification zones. In the elastic region, the walls of the pores begin to bend but are still able to recover when the load is removed. At higher loads, a plateau-like region is observed in the plastic zone, where the walls start to buckle and the porous structure starts collapsing. Finally, in the densification region, the walls are crushed and the materials behave like a non-porous material [51,52].

CNF-containing materials showed changes in mechanical properties mainly caused by the incorporation method, as well as the carboxylation of the nanofibers. For the *ex situ* preparations, it can be observed that at 3 wt.% of unmodified CNF, the values of modulus and the stress at 60% of strain began to decrease, which is attributed to the worse miscibility of unmodified fibers with polyurethane than carboxylated fiber ones, resulting in the sooner formation of fiber agglomerations [21]. The *in situ* method resulted in materials with more enhanced mechanical properties than their *ex situ* counterparts, with higher specific Young modulus and stress values. The formation of more interactions, both physical and chemical, during the synthesis process resulted in more reinforced materials. In the case of nanocomposites reinforced with the reference commercial cellulose (CNFr), lower values are measured than for systems reinforced with non-commercial nano-cellulose, ascribed to the heterogeneous cell structure formed by elongated pores, which resulted in irregular cell walls that affect the compressive properties.

Further characterization of the materials was performed, see Supplementary Information. As can be observed, materials showed high thermal stability, with all of them showing stable behavior up to 240 °C (Figure S1). It was observed that the addition of cellulose increases the thermal stability of the composites, attributed to the interactions taking place between the CNFs and the WBPUU, resulting in more stabilized urethane groups and more confined structures [1]. The DMA results also proved the effect of the addition of CNF and the incorporation route on the properties of the materials. Materials containing carboxylated cellulose nanofibers and *in situ*-prepared composites show higher storage modulus (Figure S2), owing to the higher amount of interactions formed with modified cellulose and both the physical and chemical interactions that take place using this incorporation method. Moreover, in both incorporation methods, the storage modulus is also affected by the CNF content. Moreover, mechanical α transition seen at around 40 °C in tan δ curves is delayed towards higher temperatures for the *in situ*-prepared materials, agreeing with DSC results, once again attributed to the favored polymer/reinforcements chemical and physical interactions by this incorporation method.

## 4. Conclusions

In order to prepare waterborne polyurethane–urea-based inks valid for DIW 3D printing with good final properties, the possibility of preparing and printing nanocomposite inks were analyzed. For this, different types and contents of components were employed for nanocomposite preparation, namely cellulose nanofibers and carboxylated cellulose nanofibers, which were added using two incorporation methods, *ex situ* and *in situ*. It was observed that the addition of cellulose modulated the WBPUU dispersion’s rheology, and the resulting ink’s rheological behavior was strongly dependent on the content of the CNF added. Moreover, the incorporation method also played a big role in the rheological properties of the inks, with inks prepared *ex situ* showing stronger gel-like behavior than their *in situ*-prepared homologs, due to the favored water/nano-cellulose interactions formed by this type of addition. Nonetheless, all inks showed shear-thinning behavior and most showed gel-like behavior, which allowed for their correct printability. However, an ink’s rheological properties affected the shape fidelity of the printed parts, with inks with stronger gel behavior showing higher precision. This was not only seen in the dimension of the 3D-printed parts but also in their morphology, where parts obtained from inks with lower viscosity and modulus showed collapsed porous structures.

Regarding the properties of the 3D-printed parts, the strong effect of the incorporation method of the nano-reinforcements was once again proven. The 3D-printed parts obtained from the *in situ*-prepared inks showed higher short-range interactions breaking enthalpy and temperature values, attributed to favored polymer/nano-cellulose interactions and formed chemical bonds by this incorporation method. This was also observed when studying the mechanical properties of the materials, with *in situ* parts showing higher Young modulus and stress values than the *ex situ*-prepared parts. Regarding the type of reinforcement used, it was observed that a higher amount of functional groups in carboxylated cellulose nanofibers resulted in a higher amount of interactions than untreated CNF, resulting in more reinforced materials. Overall, nanocomposite inks valid for DIW were obtained, with adjustable rheological behavior and final properties by variation of additive type, content and addition method.

## Figures and Tables

**Table 1:** Composition of WBPUU/CNF inks.

Sample	Preparation Method	Modification of CNF	Content of CNF (wt. %)	Content of PUU (wt. %)
2CNF0_EX_	*ex situ*	Untreated	2	33.9
3CNF0_EX_	*ex situ*	Untreated	3	33.6
2CNF1_EX_	*ex situ*	Carboxylated	2	33.9
3CNF1_EX_	*ex situ*	Carboxylated	3	33.6
2CNFr_IN_	*in situ*	Unknown	2	33.9
3CNFr_IN_	*in situ*	Unknown	3	33.6
3CNF0_IN_	*in situ*	Untreated	3	33.6
3CNF1_IN_	*in situ*	Carboxylated	3	33.6

**Table 2:** Viscosity and rheological parameters for *ex situ*- and *in situ*-prepared WBPUU/CNF inks.

	Sample	η at 0.2 s^−1^ (Pa·s)	$\eta \;\mathrm{at}\;{\dot{\gamma }}_{n}\;(\mathrm{Pa}\cdot s)$	η at 100 s^−1^ (Pa·s)	n
	WBPUU	1.1	0.1	0.1	0.725
*ex situ*	2CNF0_EX_	217.9	18.5	1.5	0.198
	3CNF0_EX_	434.8	18.7	1.9	0.189
	2CNF1_EX_	289.2	13.4	1.9	0.204
	3CNF1_EX_	730.6	44.5	2.7	0.111
*in situ*	2CNFr_IN_	6.2	0.9	0.1	0.361
	3CNFr_IN_	222.9	9.2	1.0	0.184
	3CNF0_IN_	256.0	14.0	2.0	0.254
	3CNF1_IN_	348.2	19.9	2.3	0.204

**Table 3:** Yield and flow point values and structure recovery capacity WBPUU/CNF inks.

	Sample	Yield Point (MPa)	Flow Point (MPa)	Structural Recovery (%)
	WBPUU	-	-	-
*ex situ*	2CNF0_EX_	16.5	89.4	78 ± 4
	3CNF0_EX_	23.6	140.0	80 ± 9
	2CNF1_EX_	15.3	59.2	75 ± 9
	3CNF1_EX_	26.6	225.6	79 ± 3
*in situ*	2CNFr_IN_	-	-	-
	3CNFr_IN_	14.5	40.1	62 ± 2
	3CNF0_IN_	14.7	61.6	67 ± 7
	3CNF1_IN_	16.5	105.1	72 ± 9

**Table 4:** Thermal properties observed from the DSC curves for 3D-printed parts obtained from WBPUU/CNF inks.

	Sample	T_g_ (°C)	T_HS_ (°C)	ΔH_HS_ (J·g^−1^)
	WBPUU	−49.1	74.7	9.0
*ex situ*	3D-2CNF0_EX_	−49.7	78.0	12.4
	3D-3CNF0_EX_	−49.0	82.0	15.5
	3D-2CNF1_EX_	−47.7	86.5	13.9
	3D-3CNF1_EX_	−48.3	81.9	16.0
*in situ*	3D-3CNFr_IN_	−50.1	77.7	15.1
	3D-3CNF0_IN_	−50.4	78.7	16.9
	3D-3CNF1_IN_	−48.4	78.0	17.6

**Table 5:** Young modulus, specific Young modulus, stress at 60% strain and densification strain values for 3D-printed parts obtained from WBPUU/CNF inks.

	Sample	Density (g·cm^−3^)	Specific Young Modulus (MPa·cm^3^·g^−1^)	Stress at 60% Strain (MPa)	Densification Strain (MPa)
*ex situ*	3D-2CNF0_EX_	0.36 ± 0.02	35.0 ± 6.0	2.8 ± 0.8	50.3 ± 0.3
	3D-3CNF0_EX_	0.36 ± 0.05	31.3 ± 3.1	2.1 ± 0.1	50.6 ± 0.5
	3D-2CNF1_EX_	0.34± 0.03	39.1 ± 8.1	2.5 ± 0.3	51.1 ± 0.5
	3D-3CNF1_EX_	0.35 ± 0.04	41.5 ± 4.0	2.5 ± 0.3	51.6 ± 0.6
*in situ*	3D-3CNFr_IN_	0.33 ± 0.02	42.5 ± 1.0	2.4 ± 0.3	51.3 ± 0.9
	3D-3CNF0_IN_	0.37 ± 0.02	56.8 ± 11.0	3.4 ±0.5	51.4 ±0.6
	3D-3CNF1_IN_	0.35 ± 0.02	59.5 ± 14.9	3.3 ± 0.6	52.0 ± 1.6

## Data Availability

The data presented in this study are available on request from the corresponding author.

## Supplementary Materials

The following supporting information can be downloaded at: https://www.mdpi.com/article/10.3390/polym14214516/s1, Figure S1: Weight evolution of freeze-dried neat WBPU and WBPU/CNF 3D printed parts; Figure S2: Storage modulus and tan δ of freeze-dried neat WBPU and WBPU/CNF 3D printed parts.
