# Supplementary material for: Effect of Cellulose Nanofibers’ Structure and Incorporation Route in Waterborne Polyurethane–Urea Based Nanocomposite Inks
Source: Polymers (Basel). 2022 Oct 25;14(21):4516. doi: 10.3390/polym14214516 (PMC9654412; doi:10.3390/polym14214516)
Supplement: Supplementary file 1 [file polymers-14-04516-s001.zip › polymers-1970563 - supplementary.pdf]

polymers

Supplementary Materials

Effect of Cellulose Nanofibers' Structure and Incorporation Route in Waterborne Polyurethane–Urea Based Nanocomposite Inks

Izaskun Larraza [1], Julen Vadillo [1,2,3], Tamara Calvo-Correas [1,4], Alvaro Tejado [5], Loli Martin [6], Aitor Arbelaiz [1,*] and Arantxa Eceiza [1,*]

1 Materials + Technologies' Research Group (GMT), Department of Chemical and Environmental Engineering, Faculty of Engineering of Gipuzkoa, University of the Basque Country, Pza Europa 1, 20018 Donostia-San Sebastian, Spain
2 IPREM-Equipe de Physique et Chimie des Polymères, UMR CNRS 5254, Université de Pau et des Pays de l'Adour, Hélioparc 2, Av. Pdt Angot, CEDEX 09, 64053 Pau, France
3 CIDETEC, Basque Research and Technology Alliance (BRTA), Po. Miramon 196, 20014 Donostia-San Sebastian, Spain
4 Department of Environmental Engineering, Faculty of Engineering of Vitoria-Gasteiz, University of the Basque Country, Nieves Cano Street, 12, 01006 Vitoria-Gasteiz, Spain
5 TECNALIA, Basque Research and Technology Alliance (BRTA), Area Anardi 5, 20730 Azpeitia, Spain
6 Macrobehavior-Mesostructure-Nanotechnology, General Research Service (SGIker), Polytechnic School, University of the Basque Country, Plaza Europa 1, 20018 Donostia-San Sebastián, Spain
* Correspondence: aitor.arbelaiz@ehu.eus (A.A.); arantxa.eceiza@ehu.eus (A.E.)

Thermogravimetric Analysis (TGA)

The thermal stability of the freeze-dried materials was determined by thermogravimetric analysis (TGA). TGA was performed in a TGA/STDA 851 (Mettler Toledo) equipment. The samples, between 5-10 mg, were heated from 30 to 700 °C in a nitrogen atmosphere at a scanning rate of 10 °C min^{-1}.

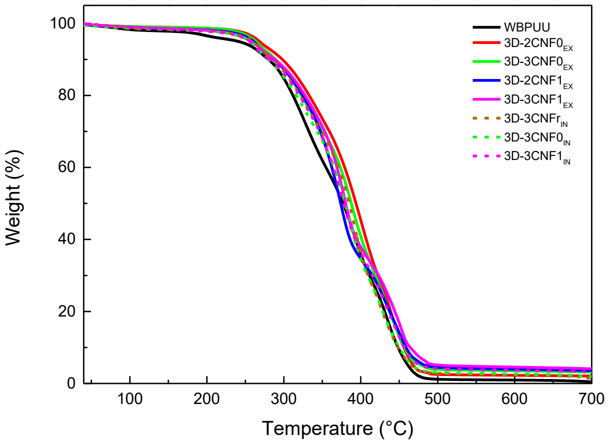

Figure S1. Weight evolution of freeze-dried neat WBPU and WBPU/CNF 3D printed parts.

Dynamic Mechanical Analysis (DMA)

The thermomechanical behavior of the 3D printed parts was determined by dynamic mechanical analysis (DMA) using an Eplexor 100 N analyzer Gabo equipment. The measurements were carried out in compression mode from -100 to 180 °C at a scanning rate of 2 °C min^{-1}. The initial strain was established as 0.3 % and the operating frequency was fixed at 1 Hz.

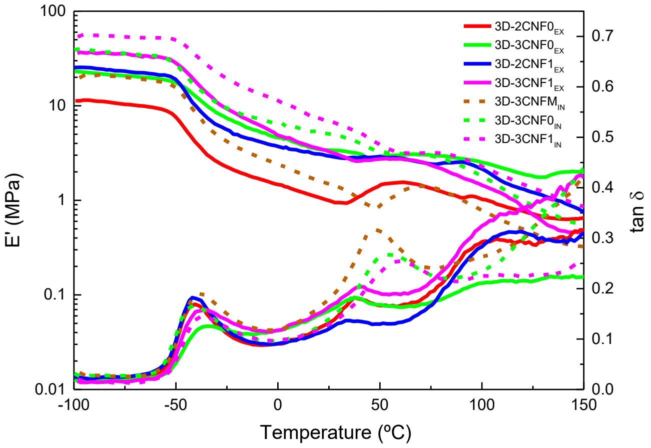

Figure S2. Storage modulus and tan δ of freeze-dried neat WBPU and WBPU/CNF 3D printed parts.
